# Adipose-Derived Stem Cell-Derived Extracellular Vesicles Inhibit the Fibrosis of Fibrotic Buccal Mucosal Fibroblasts via the MicroRNA-375/FOXF1 Axis

**DOI:** 10.1155/2021/9964159

**Published:** 2021-06-22

**Authors:** Bin Han, Yanhui Zhang, Yuxia Xiao, Bohong Shi, Hong Wu, Desheng Liu

**Affiliations:** Department of Stomatology, Rizhao People's Hospital, Rizhao, 276826 Shandong Province, China

## Abstract

Oral submucous fibrosis (OSF) is a precancerous lesion. Adipose-derived stem cell- (ADSC-) derived extracellular vesicles (EVs) (ADSC-EVs) regulate multiple oral diseases. Hence, this study explored the mechanism of ADSC-EVs in OSF. ADSCs were transduced with microRNA- (miR-) 375 mimic. ADSC-EVs and miR-375-overexpressed ADSC-EVs (EVs-miR-375) were extracted and identified. miR-375 expression in EVs and fibrotic buccal mucosal fibroblasts (fBMFs) was detected. EV uptake by fBMFs was observed. The targeted relationship between miR-375 and forkhead box protein F1 (FOXF1) was predicted and verified. After EVs-miR-375 treatment or FOXF1 overexpression, fBMF cell proliferation, migration, invasion, and apoptosis were evaluated, and levels of apoptosis-related proteins (cleaved-caspase-3, Bax, and Bcl-2) and fibrosis markers (*α*-SMA, collagen I, and collagen III) were detected. Functional rescue experiments were further performed to verify the role of the miR-375/FOXF1 axis in OSF. miR-375 was notably upregulated in EVs-miR-375 and EVs-miR-375-treated fBMFs (all *P* < 0.001). ADSC-EVs carried miR-375 into fBMFs. fBMFs can internalize ADSC-EVs. EVs-miR-375 treatment markedly inhibited fBMF cell proliferation, migration, invasion, and fibrosis and promoted apoptosis (all *P* < 0.01). Moreover, miR-375 targeted FOXF1 in fBMFs. FOXF1 overexpression promoted fBMF cell biological behaviors and fibrosis, which were reversed after EVs-miR-375 treatment (*P* < 0.01 or *P* < 0.001). We highlighted that ADSC-EVs inhibited fBMF fibrosis and then suppressed OSF progression via the miR-375/FOXF1 axis.

## 1. Introduction

Oral submucous fibrosis (OSF) is a chronic and progressive disease, which affects the whole oral cavity, pharynx, and even the esophageal mucosa [1]. As a precancerous lesion, 7%-30% of OSF evolves into malignant tumors, which seriously threaten global public health [2]. OSF is prevalent in India and Southeast Asia, where the common habits of chewing pungent food and irritants (such as betel nut and tobacco) are considered as contributing factors for OSF pathogenesis [3, 4]. From the point of the pathology, it is well accepted that excessive collagen deposition or insufficient collagen degradation in connective tissues leads to stiffness of lesion areas, thus causing restricted mouth opening [5]. Additionally, fibroblasts are indispensable participants in OSF development, since their dysfunction and fibrosis can result in hypovascularity and subsequent oral mucosa blanching, tooth and gingival staining, and trismus [6]. A previous study has proposed that fibrotic buccal mucosal fibroblasts (fBMFs) are tightly implicated in OSF [7]. However, the underlying molecular mechanism of OSF initiation and progression remains elusive, and it is of a great value to identify novel biomarkers and targets for effective therapy of OSF.

A growing number of studies have provided evidence that adipose-derived stem cells (ADSCs) are intimately involved in fibrosis in many diseases [8, 9]. Additionally, ADSCs exert therapeutic effects on oral mucosa-related diseases [10, 11]. Nevertheless, the specific roles of ADSCs in OSF are unclear. It is well established that ADSCs are able to secrete extracellular vesicles (EVs), and ADSC-derived EVs (ADSC-EVs) regulate cell function and participate in the pathology of various diseases [12].

EVs, validated as lipid-enclosed membrane structures, are promising tools for disease diagnosis and treatment [13]. EVs are reported to play a regulatory role in fibrosis of diverse organs, such as the kidney and liver [14, 15]. Mechanically, EVs can regulate the behaviors of recipient cell through the transfer of cargoes including microRNAs (miRs) [16]. A previous study has pointed out that miRs show remarkable differential expression in OSF [17]. A prior work has proposed that miR-375 overexpression in mesenchymal stem cells helps to limit the fibrosis of scar [18]. Therefore, it is reasonable to speculate that ADSC-EVs may play a role in the fibrosis of fBMFs with the involvement of miR-375 in OSF and fBMF fibrosis could be inhibited by transporting miR-375 through EVs. Consequently, we performed a series of histological and molecular experiments to identify the underlying mechanism of ADSC-EVs in OSF, with the purpose to provide some novel therapies against OSF.

## 2. Materials and Methods

### 2.1. Ethics Statement

All procedures in this study were performed with a firm compliance to the guidelines from the Institutional Review Board of Rizhao People's Hospital (Grant No. RY201810259). All patients signed informed consents.

### 2.2. Isolation and Culture of fBMFs

A total of 10 patients (8 males and 2 females; aged 30-48 years, with an average of 36.8 years) at an intermediate stage of OSF from the specialist outpatient in the department of stomatology mucosal diseases with pathological diagnosis were selected. fBMFs were isolated from buccal mucosa lesion and then primarily cultured and passaged using the tissue culture method as described in a previous study [19]. In short, the specimens were cut from the mucosa, minced, and washed twice in phosphate-buffered saline (PBS) containing antibiotics (100 U/mL penicillin, 100 mg/mL streptomycin, and 0.25 mg/mL amphotericin). Then, fBMFs were placed in a 60 mm culture dish and cultured in Dulbecco's modified Eagle's medium (DMEM, Gibco, Grand Island, NY, USA) with 10% fetal bovine serum (FBS, Gibco) and antibiotics. The fBMFs at the 3^rd^-6^th^ passage were used in the subsequent experiments.

### 2.3. Identification of fBMFs Using Immunohistochemistry

After the detachment of adherent fBMFs at the 2^nd^ passage using 0.25% trypsin, fBMFs were resuspended in DMEM containing 10% FBS and the cell density was adjusted to 2 × 10^6^ cells/mL. Cell suspension was dripped into 6-well plates with sterile slides. When cells reached over 80% confluence, the cell culture medium was discarded. The slides were removed, washed with PBS, fixed in 4% paraformaldehyde for 10-15 min, and then washed 3 times with PBS. After 10 min incubation with 0.5% Triton X-100, the cells were washed 3 times with PBS, followed by a 5 min incubation with 3% hydrogen peroxide and 3 PBS washes. Next, the cells were sealed for 10 min with goat serum at room temperature, incubated overnight with the primary antibody (pan-cytokeratin (pan-CK) (1 : 400), Vimentin (1 : 200), Boster Biological Technology Co., Ltd., Wuhan, Hubei, China) at 4°C, and rewarmed for 30 min in an incubator at 37°C. After 3 PBS washes, the cells were incubated with secondary antibody (1 : 200, ZSGB-BIO, Beijing, China) at 37°C for 15 min, developed using 2,4-diaminobutyric acid (ZSGB-BIO) for 2 min, and stained with hematoxylin (#14166, Cell Signaling Technology, Beverly, MA, USA). Finally, the slides were sealed using neutral gum and observed under an optical microscope (Olympus Optical Co., Ltd., Tokyo, Japan).

### 2.4. Identification of ADSCs

Primary ADSCs obtained from ScienCell Research Laboratories (Carlsbad, CA, USA) were cultured in an incubator at 37°C with 5% CO_2_ and saturated humidity. When the cells reached the 3^rd^ passage, flow cytometry was used to detect the expressions of stem cell surface markers (cluster of differentiation (CD) 45 (ab8216), CD31 (ab9498), CD105 (ab2529), and CD90 (ab23894)) (Abcam Inc., Cambridge, MA, USA). ADSCs at the 3^rd^ passage were cultured in osteogenic (Sigma-Aldrich, Merck KGaA, Darmstadt, Germany), adipogenic (Cyagen Biosciences, Santa Clara, CA, USA), and chondrogenic medium (Sigma-Aldrich). The differentiation potential of ADSC was analyzed using alizarin red staining (Cyagen Biosciences), oil red O staining (Sigma-Aldrich), and alcian blue glacial acetic acid staining (Shanghai Regal Biology Technology Co., Ltd., Shanghai, China) correspondingly.

### 2.5. Cell Transfection

miR-375 mimic, pcDNA3.1-forkhead box protein F1 (FOXF1), and their corresponding negative control (NC) were purchased from Guangzhou RiboBio Co., Ltd. (Guangzhou, China). ADSCs (1 × 10^5^ cells/well) in logarithmic growth phase were seeded into 6-well plates. Upon 70% confluence, the ADSCs or fBMFs were transfected using Lipofectamine 2000 (Invitrogen Inc., Carlsbad, CA, USA) according to the manufacturer's instructions.

### 2.6. Isolation and Identification of EVs

Upon 80% confluence, ADSCs were cultured in DMEM containing 10% EV-free FBS to minimize the extraneous EVs, and the supernatant was collected 48 h later. EVs were extracted from ADSC supernatant using different centrifugation and filtration procedures [20, 21]. Briefly, the cell supernatant was centrifuged twice (2000 g, 20 min; 10000 g, 40 min) successively and then filtered through a 0.22 *μ*m sterile filter (Merck Millipore Corp., Billerica, MA, USA). Next, the supernatant was centrifuged (100000 g, 70 min) and resuspended in PBS (100000 g, 70 min). To remove any residual RNA, the precipitated EVs were eluted using a mixture containing PBS and RNase I (Invitrogen). EV morphology was observed using transmission electron microscopy (TEM). ADSC-EVs were fixed in 2% paraformaldehyde for 30 min and then dripped onto the carbon-coated copper mesh. After air drying, the mixture was negative stained twice with 1% uranyl acetate. Images were captured at 120 kV using HT7700 TEM (Hitachi High-Technologies Corp., Tokyo, Japan). The size and concentration of EVs were determined using NanoSight NS300 nanoparticle tracking analysis (NTA) (Malvern Instruments, Ltd., Malvern, UK). EV surface markers CD9 (ab92726), CD63 (ab134045), and calnexin (ab22595) (Abcam) were detected using western blot (WB) analysis to characterize the ADSC-EVs.

EVs were lysed in RIPA lysis buffer, and protein concentration of EVs was detected using the Pierce bicinchoninic acid (BCA) protein detection kit (Thermo Fisher Scientific, Rockford, IL, USA) according to the manufacturer's instructions. EVs were added into the complete cell culture medium. fBMFs were treated with the culture medium containing 100 *μ*g ADSC-EVs with the conditioned medium supplemented with EV inhibitor GW4869 (MedChemExpress Co., Ltd., Monmouth Junction, NJ, USA) as control.

### 2.7. Internalization of EVs

According to the provided instructions, EVs were labeled with red fluorescent PKH26. In short, miR-375-overexpressed ADSC (EVs-miR-375) medium (100 *μ*g) was diluted in 1 mL diluent C, and PKH26 dye (4 *μ*L) was diluted in 1 mL diluent C. After gently mixing for 4 min, 2 mL 0.5% bovine serum albumin was added to bind to excessive dye, and then, the unbound dye was discarded. Next, the labeled EVs were incubated for 4 or 24 h with fBMFs. Afterwards, the cells were rinsed twice with PBS and fixed in 4% paraformaldehyde for 10 min. The nucleus was stained with 4′,6-diamidino-2-phenylindole solution (1 *μ*g/mL). The LSM 5 Exciter confocal laser scanning microscope (Carl Zeiss MicroImaging, Inc., Thornwood, NY, USA) was used to capture EV images.

### 2.8. Reverse Transcription Quantitative Polymerase Chain Reaction (RT-qPCR)

The total RNA was extracted from cells using TRIzol reagent (Invitrogen) and then reverse transcribed into cDNA using PrimeScript RT reagent kit (Takara Biotechnology Co., Ltd., Dalian, China). qPCR was carried out using SYBR® Premix Ex Taq™ II (Takara) on the ABI 7900HT fast PCR real-time system (Applied Biosystems, Foster City, CA, USA). The reaction conditions were predenaturation (95°C, 10 min) and 40 cycles of denaturation (95°C, 10 s), annealing (60°C, 20 s), and extension (72°C, 34 s). Glyceraldehyde-3-phosphate dehydrogenase (GAPDH) or U6 served as the internal control. The gene expression was analyzed using the 2^-*ΔΔ*Ct^ method. The amplified primer sequences of the genes and their primers are shown in Table 1.

### 2.9. WB（Western Blot Assay）

Protein samples were obtained using RIPA lysis buffer (strong) (Beyotime Biotechnology Co., Ltd., Shanghai, China). Protein concentration was determined using a BCA protein assay kit (Beyotime). The electrophoresis was carried out in a 4°C cold chamber for 1-2 h at an initial voltage of 60 V and then a voltage of 120 V after entering the separating gel. Then, the protein was transferred to polyvinylidene fluoride membranes via the wet electro-transfer method in a cold chamber at 4°C for 2 h. Next, the membranes were removed, blocked with 5% skim milk-tris-buffered saline-Tween 20 (TBST), and incubated for 1-2 h at room temperature. Subsequently, the membranes were placed in an incubator and added with primary antibodies for an overnight incubation at 4°C. Following 3 TBST washes (10 min each), the membranes were incubated for 1 h with horseradish peroxidase-labeled goat anti-rabbit immunoglobulin G (IgG) (1 : 5000, CoWin Biosciences, Beijing, China) at room temperature. Next, the membranes were rinsed 3 times with TBST (10 min each). The bands were developed using the chemiluminescence method, and the gray values were analyzed. GAPDH served as the internal control. The primary antibodies were anti-GAPDH (1 : 10000, ab181602, Abcam), anti-FOXF1 (1 : 1000, ab168383, Abcam), anti-B-cell lymphoma-2 (Bcl-2) (1 : 1000, ab32124, Abcam), anti-collagen I (1 : 1000, ab260043, Abcam), anti-collagen III (1 : 1000, ab184993, Abcam), anti-cleaved-caspase-3 (1 : 500, ab32042, Abcam), anti-Bcl-2-associated X (Bax) (1 : 1000, ab32503, Abcam), and anti-alpha-smooth muscle actin (*α*-SMA) (1 : 10000, ab124964, Abcam).

### 2.10. Transwell Assays

Migration and invasion were evaluated using the 24-well Transwell® system (Corning Glass Works, Corning, NY, USA) with 8 *μ*m pore filter polycarbonate membrane. The membrane was coated with Matrigel to check the invasion ability. The cells (1 × 10^5^ cells) were paved on the apical chamber in 250 *μ*L serum-free medium. The basolateral chamber was filled with medium supplemented with 10% FBS as the chemical attractant. After 48 h incubation, the filter membrane was stained with 0.1% crystal violet, and then, cells were observed under an inverted microscope (×100) and counted from 5 different fields.

### 2.11. Flow Cytometry

Cells (2 × 10^5^ cells/mL) in each group were seeded onto 12-well plates. After operations were conducted based on the instructions of Annexin V Dead Cell Kit (Beyotime), cell apoptosis was measured using a flow cytometer (Beckman Coulter, Brea, CA, USA).

### 2.12. Cell Counting Kit-8 (CCK-8) Assay

Cells (2 × 10^4^ cells/mL) in each group were seeded onto 96-well plates for 24 h. The cell viability was detected using CCK-8 assay. The corresponding volume of CCK-8 reagent was added into each well. After mixing, cells were further cultured in an incubator (37°C, 5% CO_2_) for 1.5 h. The optical density (OD) value at 450 nm was determined using a microplate reader.

### 2.13. Dual-Luciferase Reporter Gene Assay

Through database (http://www.targetscan.org/vert_71/) [22], it was predicted that there were binding sites between miR-375 and FOXF1. The complementary binding sequence and mutation sequence of miR-375 and FOXF1 were amplified and cloned into pmiR-GLO luciferase vectors (Promega, Madison, WI, USA) to construct wild-type plasmid FOXF1-WT and corresponding mutant plasmid FOXF1-MUT. FOXF1-WT or FOXF1-MUT was cotransfected with NC mimic or miR-375 mimic (Shanghai GenePharma Co., Ltd., Shanghai, China) into 293T cells using Lipofectamine™ 2000 (Invitrogen) according to the provided instructions. The luciferase activity was detected 48 h later.

### 2.14. Statistical Analysis

SPSS 21.0 (IBM Corp., Armonk, NY, USA) was used for data analysis. The Kolmogorov-Smirnov test verified data were normally distributed. The results were represented as mean ± standard deviation (SD). Comparison between two groups was analyzed using the independent sample *t*-test, and comparison among multiple groups was analyzed using one-way - analysis of variance (ANOVA), followed by Tukey's multiple comparisons test. The *P* value was obtained from a two-tailed test, where *P* < 0.05 meant a statistically significant difference.

## 3. Results

### 3.1. fBMFs and ADSCs Were Successfully Obtained

**Figure 1:**
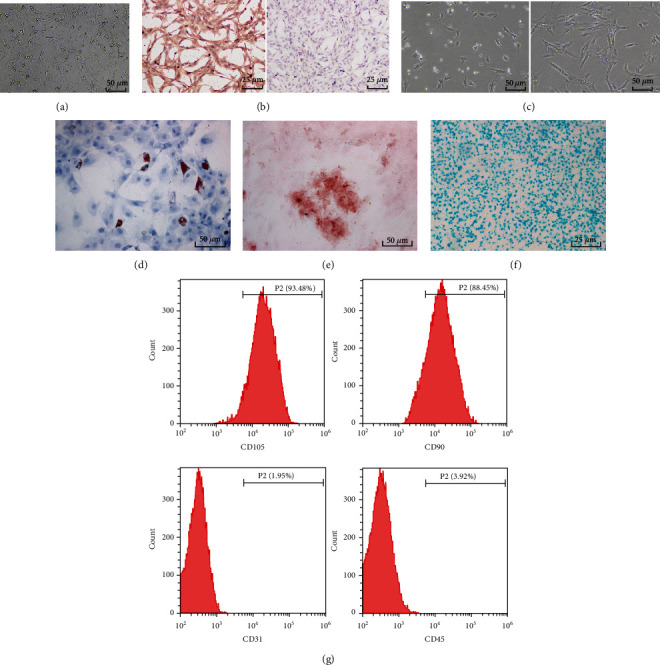
fBMFs and ADSCs are successfully obtained. The obtained fBMFs and ADSCs were identified. (a) The morphology of primary fBMFs was observed on the 5^th^ day under the microscope. (b) The expression of Vimentin and pan-CK was detected using immunohistochemistry. (c) The morphology of ADSCs was observed on the 3^rd^ and 8^th^ days of culture. (d) The adipogenic ability of ADSCs was detected using oil red O staining. (e) The osteogenic ability of ADSCs was detected using alizarin red staining. (f) The chondrogenic ability of ADSCs was detected using alcian blue glacial acetic acid staining. (g) The expression of CD105, CD90, CD45 and CD31 on the surface of ADSCs was detected using flow cytometry.

Myofibroblasts participate in collagen secretion, resulting in OSF; therefore, it is of great significance for OSF intervention and therapy to explore the targets or pathways that inhibit fBMF fibrosis [19, 23–25]. ADSCs are involved in the occurrence and development of liver fibrosis, myocardial fibrosis, and other diseases [8, 9, 26]. However, the role and mechanism of ADSCs in OSF are still unclear. In this study, fBMFs were initially extracted and identified. Briefly, on the 5^th^ day post passage and purification, the cells covered the entire bottom of the culture bottle. Cells after passages had strong proliferation ability and rapid growth rate, with basically spindle and polygonal cell morphology (Figure 1(a)). According to immunohistochemistry results, Vimentin was positively expressed and pan-CK was negatively expressed; cells were spindle or polygonal with blue-stained nucleus, brown yellow cytoplasm, and clear nuclear membrane (Figure 1(b)). From all above, it was believed that fBMFs were successfully isolated.

Next, ADSCs were identified. On the 3^rd^ and 8^th^ days of culture, we observed that most of the cells were long spindle in shape (Figure 1(c)). On the 14^th^ day of adipogenic induction, oil red O staining showed red color (Figure 1(d)), which proved that ADSCs produced fat drops. On the 21^st^ day of osteogenic induction, there was obvious calcium deposition around the cells, and red precipitate was formed in alizarin red staining (Figure 1(e)). On the 14^th^ day of chondrogenic induction, typical chondrocyte morphology and blue precipitate after staining were observed under the microscope (Figure 1(f)). These results indicated that ADSCs had osteogenic, adipogenic, and chondrogenic abilities. In addition, flow cytometry results showed that stem cell markers CD105 (93.48%) and CD90 (88.45%) were highly expressed, while hematopoietic stem cell markers CD31 (1.95%) and CD45 (3.92%) showed weak expression in ADSCs at the 3^rd^ passage (Figure 1(g)). The above results suggested that fBMFs and ADSCs were successfully obtained.

### 3.2. ADSC-EVs Were Successfully Extracted

**Figure 2:**
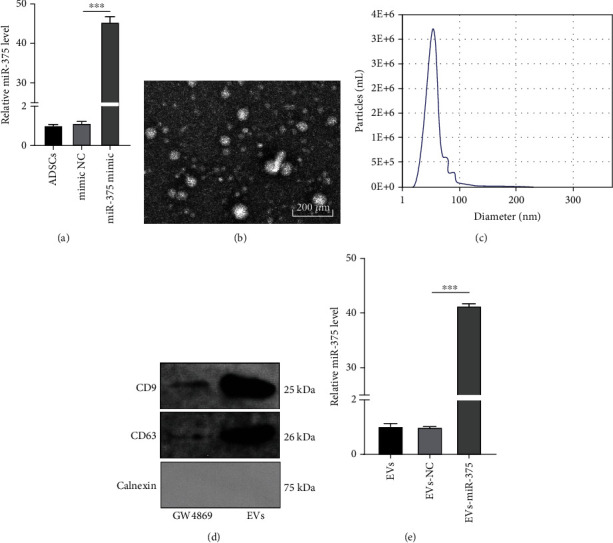
ADSC-EVs are successfully extracted. (a) ADSCs were transfected with miR-375 mimic or NC mimic, and the EVs (EVs-miR-375/EVs-NC) and EVs from ADSCs were extracted; the transfection efficiency was detected using RT-qPCR. (b) ADSC-EVs were separated and purified using ultracentrifugation methods, and their morphology and size of ADSC-EVs were analyzed under the TEM. (c) The particle size of EVs was measured using NanoSight. (d) The expression of CD9, CD63, and calnexin was detected using WB. GW4869: treatment of ADSC conditioned medium supplemented with EV inhibitor GW4869; EVs: treatment of ADSC-EVs. (e) miR-375 expression in EVs was detected using RT-qPCR. The experiment was repeated 3 times, and the data were expressed as mean ± SD. Comparison among multiple groups was analyzed using one-way ANOVA, followed by Tukey's multiple comparisons test. ^∗∗∗^*P* < 0.001.

To modify the content of EVs, ADSCs stably overexpressing miR-375 were produced by transfecting miR-375 into ADSCs, and the transfection efficiency was confirmed using RT-qPCR (*P* < 0.001) (Figure 2(a)). According to TEM observations, EVs were round cup-shaped with uniform size, and their structure was clear with a bilayer lipid membrane and a diameter of about 40-100 nm (Figure 2(b)). EV size under Brownian motion was detected using the NanoSight NTA instrument, which showed that EVs were distributed between 40 and 100 nm with a peak diameter of 55 nm (Figure 2(c)). The protein concentration was 1.5 mg/mL. WB results indicated that EV surface markers CD9 and CD63 were expressed in ADSC-EVs, while endoplasmic reticulum marker calnexin was not expressed (Figure 2(d)). Additionally, RT-qPCR revealed that compared with EVs-NC, miR-375 expression was notably increased in miR-375-overexpressed ADSC-EVs (EVs-miR-375) (*P* < 0.01) (Figure 2(e)). Altogether, ADSC-EVs were successfully extracted.

### 3.3. ADSC-EVs Were Internalized by fBMFs

**Figure 3:**
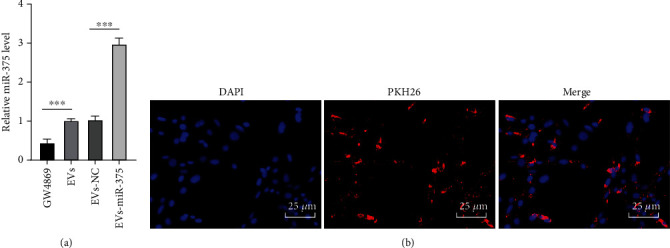
ADSC-EVs are internalized by fBMFs. fBMFs were treated with 100 *μ*g ADSC-EVs for 24 h. (a) miR-375 expression in fBMFs was detected using RT-qPCR. (b) The internalization of EVs-miR-375 by fBMFs; fBMF nuclei were labeled with DAPI (blue) and EVs-miR-375 were labeled with PKH26 (red). GW4869: treatment of ADSC conditioned medium supplemented with EV inhibitor GW4869; EVs: treatment of ADSC-EVs; EVs-miR-375/EVs-NC: treatment of EVs derived from ADSCs transfected with miR-375 mimic or NC mimic. The experiment was repeated 3 times, and the data were expressed as mean ± SD. Comparison among groups was analyzed using one-way ANOVA followed by Tukey's multiple comparisons test. ^∗∗∗^*P* < 0.001.

After fBMFs were treated with 100 *μ*g ADSC-EVs for 24 h, miR-375 was notably upregulated in fBMFs and also elevated in EVs-miR-375-treated fBMFs, as shown by RT-qPCR results (all *P* < 0.001) (Figure 3(a)). According to confocal laser scanning microscope observations, PKH26-labeled EVs (red dots) were gradually internalized by fBMFs (Figure 3(b)). From above, we confirmed that the ADSC-EV-carried miR-375 mediated the communication between ADSCs and fBMFs and may participate in regulating fBMF functions.

### 3.4. miR-375-Overexpressed ADSC-EVs Inhibited the Fibrosis of fBMFs

**Figure 4:**
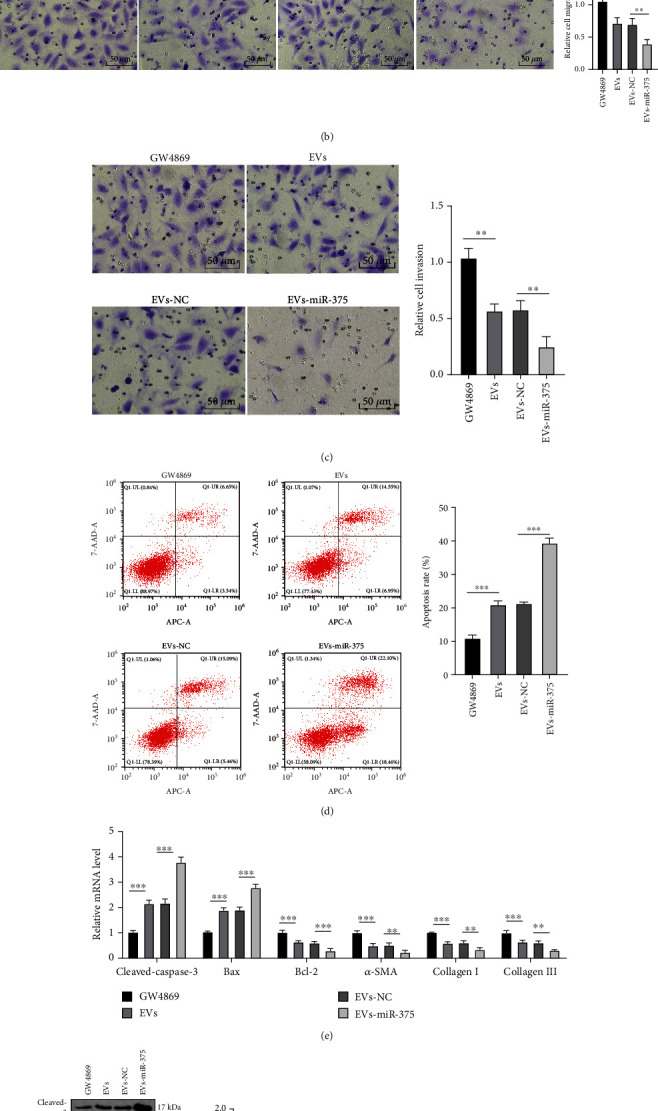
miR-375-overexpressed ADSC-EVs inhibit the fibrosis of fBMFs. fBMFs were treated with EVs or miR-375-overexpressed ADSC-EVs (EVs-miR-375) or the corresponding controls. (a) The proliferation of fBMFs was detected using CCK-8 assay. (b, c) The migration and invasion of fBMFs were detected using Transwell assays. (d) The apoptosis of fBMFs was detected using flow cytometry. (e, f) Levels of proapoptotic proteins (cleaved-caspase-3 and Bax), antiapoptotic protein Bcl-2, and fibrosis markers (*α*-SMA, collagen I, and collagen III) were detected using RT-qPCR and WB. The experiment was repeated 3 times, and the data were expressed as mean ± SD. Comparison among groups was analyzed using one-way ANOVA, followed by Tukey's multiple comparisons test. ^∗∗^*P* < 0.01 and ^∗∗∗^*P* < 0.001.

To further study whether ADSC-EV-carried miR-375 is involved in the regulation of fBMF fibrosis, EVs-miR-375 were cultured with fBMFs to explore the function changes of fBMFs and expressions of fibrosis- and apoptosis-related proteins and collagen. CCK-8 assay showed that EV-treated fBMFs showed notably decreased proliferation ability, and EVs-miR-375 treatment further reduced the fBMF cell proliferation (both *P* < 0.01) (Figure 4(a)). According to Transwell assays, EV treatment inhibited fBMF cell migration and invasion, which were further suppressed after EVs-miR-375 treatment (both *P* < 0.01) (Figures 4(b) and 4(c)). Flow cytometry results revealed that the apoptotic rate of EV-treated fBMFs was elevated, and EVs-miR-375 treatment caused a more notably increased apoptosis rate (both *P* < 0.001) (Figure 4(d)). In addition, RT-qPCR and WB results suggested that EV treatment promoted the levels of proapoptotic proteins (cleaved-caspase-3 and Bax) and inhibited the levels of antiapoptotic protein Bcl-2 and fibrosis markers (*α*-SMA, collagen I, and collagen III). EVs-miR-375 treatment strengthened the role of EVs (all *P* < 0.01) (Figures 4(e) and 4(f)). Briefly, miR-375-overexpressed ADSC-EVs inhibited the fibrosis of fBMFs.

### 3.5. miR-375 Targeted FOXF1 in fBMFs

**Figure 5:**
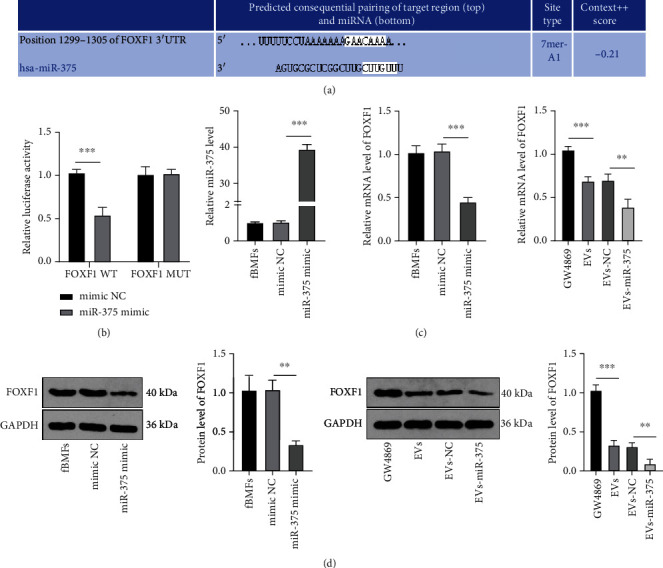
miR-375 targets FOXF1 in fBMFs. (a) The binding sites of miR-375 and FOXF1 were analyzed. (b) Dual-luciferase reporter gene assay was used to detect the binding relationship between miR-375 and FOXF1. (c, d) The expression of FOXF1 in each group was detected using RT-qPCR and WB. The experiment was repeated 3 times, and the data were expressed as mean ± SD. Comparison among groups was analyzed using one-way ANOVA, followed by Tukey's multiple comparisons test. ^∗∗^*P* < 0.01 and ^∗∗∗^*P* < 0.001.

Through database (http://www.targetscan.org/vert_71/) [22], it was predicted that there were binding sites between miR-375 and FOXF1 (Figure 5(a)), and their targeted binding relationship was verified using dual-luciferase reporter gene assay (*P* < 0.001) (Figure 5(b)). To further explore the regulatory effect of miR-375 on FOXF1, miR-375 mimic was transfected into fBMFs (*P* < 0.001) (Figure 5(c)). As shown by RT-qPCR and WB results, miR-375 overexpression remarkably inhibited FOXF1 expression in fBMFs (all *P* < 0.01) (Figures 5(c) and 5(d)). FOXF1 expression was also noticeably suppressed after EV and EVs-miR-375 treatment (all *P* < 0.01). These results suggested that FOXF1 was a target gene of miR-375, and EV-carried miR-375 negatively regulated FOXF1 expression.

### 3.6. EV-Carried miR-375 Inhibited the Fibrosis of fBMFs via Targeting FOXF1

**Figure 6:**
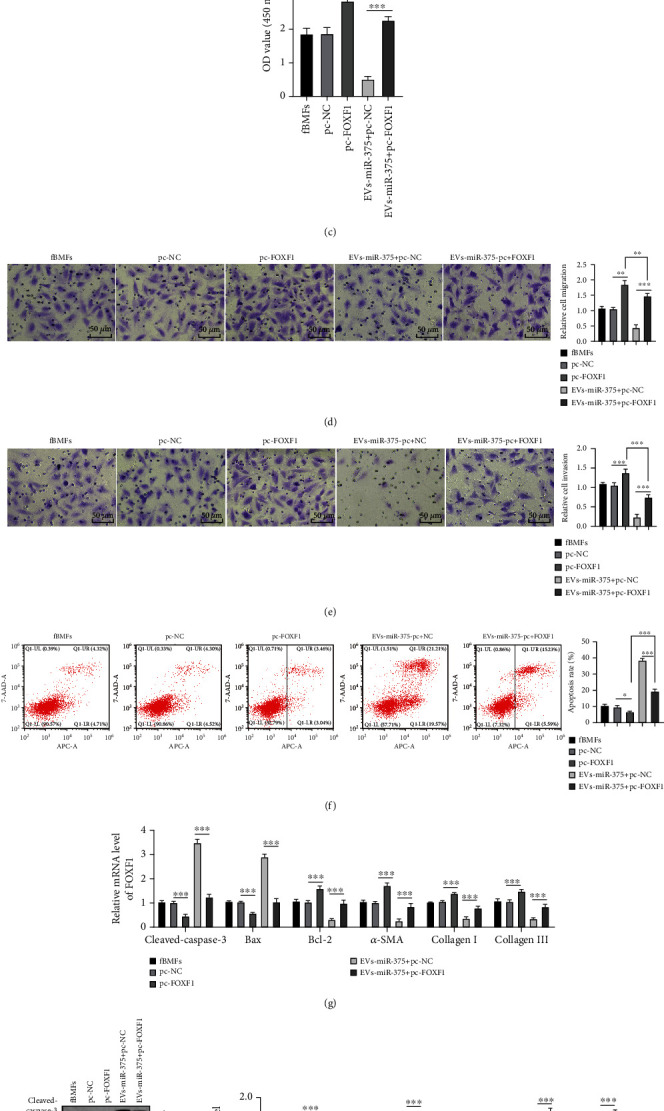
EV-carried miR-375 inhibits the fibrosis of fBMFs via targeting FOXF1. EVs-miR-375 and plasmid overexpressing FOXF1 (pc-FOXF1)/pc-NC were transfected into fBMFs. (a, b) The successful transfection of FOXF1 was verified using RT-qPCR and WB. (c) CCK-8 assay was used to detect the proliferation of fBMFs. (d, e) Transwell assay was used to detect the migration and invasion of fBMFs. (f) Flow cytometry was used to detect the apoptosis of fBMFs cells. (g, h) RT-qPCR and WB were used to detect the levels of proapoptotic proteins (cleaved-caspase-3 and Bax), antiapoptotic protein Bcl-2, and fibrosis markers (*α*-SMA, collagen I, and collagen III). pc-NC/pc-FOXF1: fBMFs transfected with pc-NC/pc-FOXF1; EVs-miR-375+pc-NC/EVs-miR-375+pc-FOXF1: combined treatment of EVs-miR-375 and pc-NC/pc-FOXF1 in fBMFs. The experiment was repeated 3 times, and the data were expressed as mean ± SD. Comparison among groups was analyzed using one-way ANOVA, followed by Tukey's multiple comparisons test. ^∗∗^*P* < 0.01 and ^∗∗∗^*P* < 0.001.

To identify whether EV-carried miR-375 regulates the fibrosis of fBMFs through FOXF1, plasmids overexpressing FOXF1 (pc-FOXF1) or pc-NC were transfected into fBMFs treated with EVs-miR-375. The transfection of FOXF1 was verified using RT-qPCR and WB (both *P* < 0.001) (Figures 6(a) and 6(b)). CCK-8 assay suggested that FOXF1 overexpression obviously promoted cell proliferation, which was notably inhibited after EVs-miR-375 treatment (all *P* < 0.01) (Figure 6(c)). As shown by Transwell assays, FOXF1 overexpression notably increased fBMF cell migration and invasion, while EVs-miR-375+pc-FOXF1 treatment caused the opposite results (all *P* < 0.01) (Figures 6(d) and 6(e)). Flow cytometry revealed that the apoptosis rate of fBMFs was remarkably reduced in pc-FOXF1-treated fBMFs, which was then obviously elevated after EVs-miR-375 treatment (all *P* < 0.001) (Figure 6(f)). According to RT-qPCR and WB results, FOXF1 overexpression clearly decreased the levels of proapoptotic proteins (cleaved-caspase-3 and Bax) and elevated the levels of antiapoptotic protein Bcl-2 and fibrosis markers (*α*-SMA, collagen I, and collagen III), while EVs-miR-375+pc-FOXF1 treatment reversed the above trends (all *P* < 0.01) (Figures 6(g) and 6(h)). Collectively, EV-carried miR-375 inhibited the fibrosis of fBMFs via targeting FOXF1.

## 4. Discussion

OSF may evolve to malignant tumors and greatly affects patient's quality of life [27]. It has shown that ADSC-EVs help to reduce the fibrosis in mice [28]. The present study elucidated that ADSC-EVs inhibited fBMF fibrosis, thereby suppressing OSF progression via the miR-375/FOXF1 axis.

Studies have provided overwhelming evidence that ADSCs are crucial in the fibrosis in various diseases [8, 26]. ADSC can secrete EVs and ADSC-EVs have strong potential for disease therapies [12]. ADSC-EVs show protective effects against myocardial and renal fibrosis [9, 29]. Moreover, a previous study proved that myofibroblast activities in fBMFs are strongly associated with OSF progression [30]. Local stem cells can biologically act as the immunomodulatory and proregenerative activities in the local environment [31]. Local stem cells may exert the same effect but lack the advantages of MSCs in other perspectives. For instance, the applications of dental-derived stem cells are limited for reasons such as difficulty in obtaining and that is the reason why other stem cells were not used as seed cells in this study. Therefore, following identifications of ADSCs, fBMFs, and ADSC-EVs, we investigated the role of ADSC-EVs in fBMF fibrosis in OSF. EVs encapsulate multiple functional molecules including miRs to play pathobiological roles in diverse diseases [32]. Studies have demonstrated that miR-375 is tightly implicated in the fibrosis of multiple organs, such as the liver and intestine [33, 34]. However, little is known about the role of EV-carried miR-375 in the fBMF fibrosis in OSF. Thereafter, we initially constructed miR-375-overexpressed ADSCs and found that miR-375 expression in miR-375-overexpressed ADSC-EVs (EVs-miR-375) and EVs-miR-375-treated fBMFs was notably increased. Likewise, as has been pointed out previously, decreased miR-375 expression in oral premalignant lesions is closely linked to high risks of malignant transformation [35]. ADSC-derived exosomes deliver miR-375 into recipient cells to promote bone regeneration [36]. Briefly, ADSC-EVs carried miR-375 into fBMFs.

Next, the specific role of ADSC-EV-carried miR-375 in fBMFs was investigated. Compelling evidence suggests that the proliferation, migration, and myofibroblast activities of fBMFs are intrinsically associated with OSF pathogenesis [19, 23–25]. Principally, *α*-SMA, collagen I, and collagen III are potent fibrosis markers [37]. According to our results, miR-375 overexpression in ADSCs notably strengthened the role of EVs, as evidenced by decreased proliferation, migration, and invasion, elevated apoptosis of fBMFs, and inhibited levels of fibrosis markers (*α*-SMA, collagen I, and collagen III). In support of these, miR-375 overexpression in mesenchymal stem cells inhibits the transition of fibroblasts into myofibroblasts, thus inhibiting fibrosis in mouse scar region [18]. miR-375 suppresses cell proliferation of arthritis synovial fibroblasts [38]. Furthermore, a previous study has proposed that miR-375 may show a close relation to oral mucosa-related diseases [39]. Taken together, miR-375-overexpressed ADSC-EVs inhibited the fibrosis of fBMFs.

Subsequently, we aimed to investigate the downstream mechanism of miR-375 in fBMF fibrosis in OSF. Through prediction and dual-luciferase assay, we identified that FOXF1 was the target gene of miR-375; FOXF1 expression in fBMFs was remarkably inhibited after transfection of miR-375 mimic or treatment of EVs/EVs-miR-375. Similarly, a previous study supports the targeted relationship between miR-375 and FOXM1, a member of the forkhead box family like FOXF1 [40]. Briefly, ADSC-EV-carried miR-375 targets FOXF1 expression in fBMFs. Next, to verify the role of the miR-375/FOXF1 axis in regulating fBMF fibrosis in OSF, we overexpressed FOXF1 in fBMFs. Our findings elicited that FOXF1 overexpression obviously promoted cell proliferation, migration, and invasion and fibrosis marker levels and reduced apoptosis in fBMFs, while EVs-miR-375 treatment reversed all the above trends. Consistently, a previous work has demonstrated that FOXF1 expression in myofibroblasts is tightly associated with fibrotic lesions [41]. Nevertheless, the interplay between miR-375 and FOXF1 in the fibrosis of fBMFs in OSF remains elusive, which implies the novelty of this study. In summary, EV-carried miR-375 inhibited the fibrosis of fBMFs via targeting FOXF1.

All in all, this study found that ADSC-EV-shuttled miR-375 inhibited fBMF fibrosis and alleviated OSF by targeting FOXF1. ADSC-EV-based medical interventions may be effective approaches for OSF therapy. miR-375 and FOXF1 are promising targets for OSF treatment. In the future, we will explore the role and regulatory mechanism of ADSC-EV-carried miR-375 in OSF at the animal level. Additionally, the combination of highly porous, biologically active (biointeractive) scaffolds with autologous human periapical cyst mesenchymal stem cells may be a promising tool for regenerative healing in dentistry [42]. The mechanical stimuli regulating the behavior and function of dental pulp stem cells indicate a prospect in the regeneration of bone and dental tissues [43]. Hence, overall effects of specific novel and interesting biomaterials on cell growth and behaviors require further exploration as well. Tissues and clinical inflammation and injury are also critical [44, 45]. However, so far, the research on animal experiment and tissue inflammation cannot be conducted due to the pandemic. Here, the effect of miR-374 in ADSCs-EVs on fBMF fibrosis was the major intention. We will study on tissue inflammation in the future. Although the present study provides therapeutic value for OSF treatment, the experiment results and clinical application need to be further verified.

## Figures and Tables

**Table 1:** Primer sequences for RT-qPCR.

Gene	Primer
miR-375	F: 5′-CAAAGTGCTTACAGTGCAGGTAG-3′
	R: 5′-CTACCTGCACTGTAAGCACTTTG-3′
FOXF1	F: 5′-ATGTCTTCGGCGCCCGAGAAGCAGC-3′
	R: 5′-TCACATCACGCAAGGCTTGATGTCT-3′
Bcl-2	F: 5′-ATGGCGCACGCTGGGAGAACAGGG-3′
	R: 5′-TCACTTGTGGCCCAGATAGGCACC-3′
Collagen I	F: 5′-ATGTTCAGCTTTGTGGACCTCCG-3′
	R: 5′-TTACAGGAAGCAGACAGGGCCAA-3′
Collagen III	F: 5′-ATGATGAGCTTTGTGCAAAAGGG-3′
	R: 5′-TTATAAAAAGCAAACAGGGCCAAC-3′
Cleaved-caspase-3	F: 5′-ATGGAGAACACTGAAAACTCAGT-3′
	R: 5′-TTAGTGATAAAAATAGAGTTCTT-3′
Bax	F: 5′-ATGGACGGGTCCGGGGAGCAGCCCA-3′
	R: 5′-TTATGGAGGAAAAACACAGTCCAAG-3′
*α*-SMA	F: 5′-TAGAAGCATTTGCGGTGGACAATG-3′
	R: 5′-GCAGCTCTAGGAGCATGTGG-3′
U6	F: 5′-CGCTTCGGCAGCACATATAC-3′
	R: 5′-AATATGGAACGCTTCACGA-3′
GAPDH	F: 5′-ATGGTTTACATGTTCCAATATGA-3′
	R: 5′-TTACTCCTTGGAGGCCATGTGG-3′

## Data Availability

All the data generated or analyzed during this study are included in this published article.
